# Examining Type 1 Diabetes Mathematical Models Using Experimental Data

**DOI:** 10.3390/ijerph19020737

**Published:** 2022-01-10

**Authors:** Hannah Al Ali, Alireza Daneshkhah, Abdesslam Boutayeb, Zindoga Mukandavire

**Affiliations:** 1 Computational Science and Mathematical Modelling, Coventry University, Coventry CV1 5FB, UK; ac5916@coventry.ac.uk; 2 Institute of Applied Research and Technology, Emirates Aviation University, Dubai 53044, United Arab Emirates; zindoga.mukandavire@emirates.com; 3 Centre for Data Science and Artificial Intelligence, Emirates Aviation University, Dubai 53044, United Arab Emirates; 4 Department of Mathematics, Faculty of Sciences, University Mohamed Premier, P.O. Box 524, Oujda 60000, Morocco; x.boutayeb@gmail.com

**Keywords:** diabetes, thresholds, model selection, model calibration

## Abstract

Type 1 diabetes requires treatment with insulin injections and monitoring glucose levels in affected individuals. We explored the utility of two mathematical models in predicting glucose concentration levels in type 1 diabetic mice and determined disease pathways. We adapted two mathematical models, one with β-cells and the other with no β-cell component to determine their capability in predicting glucose concentration and determine type 1 diabetes pathways using published glucose concentration data for four groups of experimental mice. The groups of mice were numbered Mice Group 1–4, depending on the diabetes severity of each group, with severity increasing from group 1–4. A Markov Chain Monte Carlo method based on a Bayesian framework was used to fit the model to determine the best model structure. Akaike information criteria (AIC) and Bayesian information criteria (BIC) approaches were used to assess the best model structure for type 1 diabetes. In fitting the model with no β-cells to glucose level data, we varied insulin absorption rate and insulin clearance rate. However, the model with β-cells required more parameters to match the data and we fitted the β-cell glucose tolerance factor, whole body insulin clearance rate, glucose production rate, and glucose clearance rate. Fitting the models to the blood glucose concentration level gave the least difference in AIC of 1.2, and a difference in BIC of 0.12 for Mice Group 4. The estimated AIC and BIC values were highest for Mice Group 1 than all other mice groups. The models gave substantial differences in AIC and BIC values for Mice Groups 1–3 ranging from 2.10 to 4.05. Our results suggest that the model without β-cells provides a more suitable structure for modelling type 1 diabetes and predicting blood glucose concentration for hypoglycaemic episodes.

## 1. Introduction

Diabetes, a global epidemic, has two main forms—type 1 and type 2 diabetes. Approximately 463 million adults were living with diabetes in 2021, and this is expected to rise to 700 million by 2045 [1]. The disease can affect any individual, regardless of size, age, or gender, and there are many factors that can increase risk of having diabetes. Diabetes can be considered as the irregularities in the glucose homeostasis system where homeostasis is not able to be maintained or controlled [2]. Symptoms of all forms of diabetes are, increased thirst, urination, hunger, tiredness, weight lost, and blurred vision [2]. The severity of the disease arises when complications appear. Complications of diabetes are heart disease, kidney failure, nerve damage, comas, and eventually death [3,4]. A recent concern of diabetes is related to the COVID-19 pandemic. COVID-19 is known for attacking the immune system and individuals with diabetes are extremely vulnerable to contracting the highly infectious virus, having already compromised immune systems. Several studies have linked diabetes to increased severity of COVID-19 infection and hindering quick recovery [5,6].

Type 1 diabetes (i.e., insulin dependent diabetes) usually occurs from a young age and is classified as an autoimmune disease. Type 1 diabetes occurs due to an autoimmune reaction from the body, destroying the β-cells in an individual [7]. Without β-cells, an individual cannot produce insulin, which is required to reduce blood glucose levels back within the normal range [2]. Without control of the blood glucose levels, diabetes complications arise, and eventually will lead to death [2]. Treatment and management of type 1 diabetes requires daily injections of insulin and constant blood glucose monitoring, healthy diet, and exercise [7].

Type 2 diabetes (i.e., non-insulin dependent diabetes) is present more frequently in individuals who are overweight and 80% of individuals diagnosed with type 2 are overweight [8]. Type 2 diabetes is reversible and can develop over time if risk factors, such as being overweight, unhealthy diet, and high blood pressure manifest; it constitutes 90% of diabetes cases [1]. Unlike type 1 diabetes, individuals with type 2 diabetes do have β-cells present, but insufficient insulin secretion to control glucose levels [2]. This is caused by insulin resistance, which occurs when the body produces insulin, but fails to effectively use it [9]. The body therefore becomes resistant to its own insulin and attempts to compensate by producing higher quantity of insulin [7]. This in turn leads to β-cells wearing out or β-cell burnout [7]. Management of type 2 diabetes takes places through diet, exercise, and a healthy lifestyle, although oral medications may be given if individuals are not able to control their glucose levels through diet and exercise [2]. In some cases, insulin injections may also be given; however, this is usually administered to individuals with type 1 diabetes [10].

Treatment and management of diabetes is becoming increasingly important especially within the current COVID-19 pandemic. Most of type 2 diabetes cases can be reversed and nearly most prevented, but type 1 diabetes is irreversible; thus, treatment and management of the disease in this form is very important. In 2020, more than 1.1 million children and adolescents were living with type 1 diabetes globally [1]; thus, increasing the urgent need to establish efficient treatment and management strategies of the disease. Although manageable, the major challenge with diabetes involves the fatal complications that can occur if not properly managed [3,4].

Previous mathematical models of the glucose homeostasis system, which were confronted with data, considered models with different components, such as the amount of glucose in the digestive system as a separate entity and the amount of glucose present in urine [11,12]. In our models, studied in [3,13], we hypothesized that glucose concentration in the system is accounted for in one equation rather than breaking down the glucose concentration in different parts of the body and separating it into different equations. Other glucose homeostasis models that fit to the data are based on models in which plasma insulin and glucose levels are considered as separate equations to exogenous insulin and the effect of insulin on glucose levels [14]. Delay differential equations, consisting of a system of equations to model different parts of insulin, glucose concentration, and plasma levels were used to model diabetes and fitted to data in [15]. Further, the use of time delays is common in modelling the glucose–insulin regulatory system [16,17,18,19] because of the need to consider a time delay in insulin secretion in response to elevated blood glucose concentration.

Development of suitable mathematical models to understand glucose homeostasis system in a diabetic individual is urgent and key in developing automatic insulin pumps for disease treatment, management, and control. Several mathematical models are used as algorithms in continuous glucose monitoring devices, insulin pumps, and an artificial pancreas [20,21,22], and these devices require accurate predictive models. Model comparison and selection play important roles in identifying the best model from a set of candidate models for data-driven modelling and system identification problems [23]. Existing diabetes models for type 1 and type 2 include β-cells [3,8,11,18,24,25,26,27,28,29,30,31]. In this study, we adapted two mathematical models from our previous work, one with β-cells [13] and one without the β-cell component [3], to determine their capabilities in predicting blood glucose concentration levels and identifying type 1 diabetes pathways using published experimental data from mice studies [32]. In [32], these data were fitted to a model that partly considered glucose in the digestive system, but in our case, we explored the performance of the two models for whole body glucose dynamics to understand diabetes pathways. Both models describe the glucose homeostasis system of an individual, glucose, insulin, and growth hormone concentration levels in an individual. The main difference between the models is that one has a β-cells component and the other has no β-cells, as it was theoretically designed to describe the glucose homeostasis system of a purely type 1 diabetic individual. Type 1 diabetics have zero or relatively low β-cells; thus, we assume that the second model has no β-cell component [7] in order to adequately capture this characteristic. This study is the first attempt to use data-driven approaches to generate evidence on the utility of these models in predicting blood glucose concentration levels in type 1 diabetics and in disease pathways. The penalised model selection approach is commonly used for model comparison and selection. In this study, we use the Akaike information criterion [33] and Bayesian information criterion [34], which are common penalised model selection criteria used in several disciplines.

## 2. Methods

### 2.1. Data

**Figure 1:**
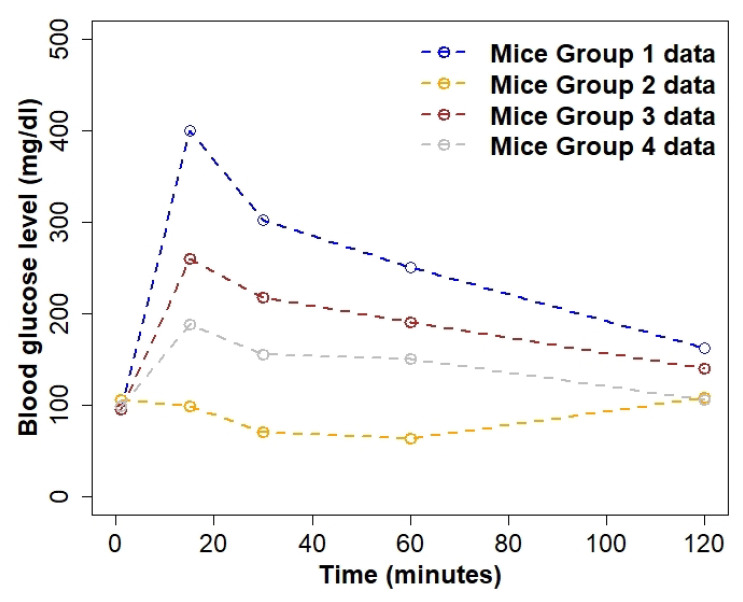
Glucose concentration data for four groups of experimental mice extracted from [32]. The blue line represents Mice Group 1 glucose concentration level data; Mice Group 2 is denoted by the orange line; Mice Group 3 is denoted by the dark red line; and Mice Group 4 is denoted by the grey line.

We used published data on mean blood glucose concentration levels for four small experimental groups of mice (i.e., sample sizes of 5–6) [32]. The four groups of mice used in this study were, from various times, exposed to bisphenol S, a chemical that hinders glucose homeostasis in individuals and accelerates type 1 diabetes [32]. Further details on the mice used and their protocols can be found in [32]. Mathematical models to predict diabetes pathways in humans are usually tested using data from experimental studies in rodents [35]. Many of the studies are in vitro but it is also essential to have in vivo studies; hence, rats and mice are the most commonly used animals to study the glucose homeostasis system [35]. Most importantly, glucose homeostasis in humans and rodents is maintained by the same factors (i.e., finding the balance between glucose and insulin concentration in the blood); hence, it is acceptable to use data from rodents and apply the results to understand diabetes dynamics in humans [36]. The data are based on four groups of experimental mice with diabetes, were manually extracted from [32], and are presented in Figure 1. We numbered the four groups as Mice Groups 1–4, depending on diabetes severity, with Mice Group 1 having a hyperglycaemic episode peaking at 400 mg/dL (data extracted from Figure 1D in [32]) and Mice Group 4 (data extracted from Figure 3D in [32]) maintaining glucose concentration within the expected ranges of 70–200 mg/dL [4]. The blood glucose data for all four groups of mice in Figure 1 illustrate some biological characteristics of the groups of mice. Mice Group 1 had a hyperglycaemic episode with extremely high levels of glucose concentration appearing rapidly. In Figure 1, Mice Group 2 (data extracted from Figure 3C in [32]) started within normal glucose concentration ranges and rapidly became hypoglycaemic (glucose concentration is dangerously low). This suggests that Mice Groups 1 and 2 may not have been able to control their glucose homeostasis systems. Mice Group 1 showed a hyperglycaemic episode, where excess glucose was in the blood, but not enough insulin was injected to control it. On the other hand, Mice Group 2 showed a hypoglycaemic episode, where the glucose levels were too low in the blood and less insulin needed to be injected to control the system. Mice Group 1 likely experienced several symptoms, such as nausea, dizziness, feeling faint, weakness, and possibly death if the glucose levels did not decrease to within normal ranges [4]. Similarly, Mice Group 2 likely experienced the same severe symptoms; remaining in dangerously low levels of glucose and death could occur if the levels of glucose do not normalise [4]. We note that Mice Group 3 (data extracted from Figure 1D in [32]), although diabetic, was within expected blood glucose concentration ranges (<260 mg/dL) [4] and showed slightly higher levels than that of non-diabetics. Mice Group 4 appears to have been within the expected glucose concentration ranges, showing the glucose homeostasis system to be under good control.

### 2.2. Diabetes Candidate Models

We use two mathematical models, which represent the pathways to diabetes, one specifically describes type 1 diabetes [3] and another that can represent both forms of diabetes (type 1 and type 2) [13]. Models 1 and 2 simulate the insulin concentration, glucose concentration, and growth hormone concentration in an individual and their formulation details are presented in [3,13], respectively. The models were parameterised using experimental data from mice studies, which were extracted from published literature [24,32]. Models 1 and 2 are respectively given in the model system (1) and (2), their model input parameters and response variables are presented in Table 1. Both models are based on the minimal model, which was proposed by [25,37,38] and is widely used in the intravenous glucose tolerance test.

Model 1 (a type 1 diabetes model) consists of the following response variables, insulin (I), glucose $\left(G_{L}\right)$, and growth hormone $\left(G_{H}\right)$ and incorporates an insulin injection term, $I_{0}$. Here, we briefly describe the Model 1 formulation details presented in [3]. The model does not consider β-cell dynamics and incorporates a subcutaneous insulin injection term, $I_{0}$, which represents a constant bolus value. The insulin levels in the blood are a product of the amount of insulin externally injected and the absorption rate, ψ. The insulin injection term, $I_{0}$, is hypothesised to have an inversely proportional relationship with insulin concentration in the blood (I) [39,40,41,42]. We model this relationship using the term $\frac{I_{0}}{1+I}$. Overtime blood insulin level drops as glucose is absorbed by muscle, fat, and liver cells, and clears at a constant rate δ. Glucose $\left(G_{L}\right)$ levels are increased by the growth hormone through suppression of glucose uptake by insulin, at a constant rate *c*. The parameter *a* represents total glucose production rate in the liver. The growth hormone $\left(G_{H}\right)$ is increased by the rate of production by the somatotropic cells in the pituitary gland at a constant rate ρ and decreases at a rate *w* through absorption by the liver [43]. Model 1 is governed by the following system of differential equations [3].

(1) $$\left.\begin{array}{c} \frac{dI}{dt}=\frac{\psi I_{0}I}{1+I}-\delta I,\\ \frac{dG_{L}}{dt}=a-\left(b+cI\right)G_{L}+cG_{H},\\ \frac{dG_{H}}{dt}=\rho -wG_{H}. \end{array}\right\}$$

Model 2 is the glucose homeostasis model and consists of β-cells (β), insulin (I), glucose $\left(G_{L}\right)$, and growth hormone $\left(G_{H}\right)$. Here, we briefly describe Model 2 formulation details presented in [13]. The β-cells increase, by production or replication at rate *h*, and are reduced by β-cell exhaustion or natural death at a rate *g*. Insulin (I) is secreted by the β-cells and is dependent on the levels of glucose in the body. Insulin is reduced by natural clearance at a rate *f*. The net insulin rate was determined to be best modelled as a sigmoidal function of the glucose level [24]. Glucose $\left(G_{L}\right)$ levels are increased by the growth hormone by suppressing glucose uptake by insulin, at a constant rate *c*. Parameter *a* represents the total glucose production rate in the liver. The growth hormone $\left(G_{H}\right)$ is increased by the rate of production by the somatotropic cells in the pituitary gland at a constant rate ρ. $G_{H}$ is decreased at a rate *w* through absorption by the liver [43]. Model 2 is governed by the following system of differential equations [13].

(2) $$\left.\begin{array}{c} \frac{d\beta }{dt}=\left(hG_{L}-iG_{L}^{2}-g\right)\beta ,\\ \frac{dI}{dt}=\frac{\beta dG_{L}^{2}}{e+G_{L}^{2}}-fI,\\ \frac{dG_{L}}{dt}=a-\left(b+cI\right)G_{L}+cG_{H},\\ \frac{dG_{H}}{dt}=\rho -wG_{H}. \end{array}\right\}$$

The model parameters and variables of both models are presented in Table 1 and the parameter values and variable estimates were extracted from the published literature [3,8,10,43,44,45], and other parameter values were assumed.

### 2.3. Model Calibration

We fitted the models to experimental data on mice published in [32]. We manually extracted mean values of blood glucose concentration levels from the figures presented in [32]. We considered the mean blood glucose concentration to be more suitable to use for the purposes of our study. A Markov Chain Monte Carlo (MCMC) based on a Bayesian framework was used to fit the diabetes models to blood glucose concentration level data. The flexible modelling environment (FME) package in R [46], was used to implement an MCMC algorithm based on the delayed rejection and adaptive Metropolis procedure [47]. A similar approach was also applied in modelling infectious diseases [48,49]. We systematically varied the parameters that were previously shown to be influential through a mathematical analysis conducted in [3] and used these to guide our fitting process for each model. In fitting Model 1, we varied insulin clearance rate (δ) and insulin absorption rate (ψ). We deduced through a systematic analysis that Model 2 required more parameters to be varied in order to fit the glucose concentration data and we varied glucose production rate (*a*), glucose clearance rate (*b*), whole body insulin clearance rate (*f*), and the β-cell glucose tolerance range factor (*i*).

In the fitting, glucose concentration ($G_{L}$) was estimated by varying parameters, *a*, δ, *b*, ψ, *b*, *f* and *i*, whilst other parameters remained fixed as given in Table 1. We assumed a uniform prior for each varied parameter and parameters were sampled within lower and upper values of assumed values and published literature given in Table 1. When fitting the models to blood glucose levels, we assumed the observations to be identically and independently distributed with additive Gaussian noise and unknown variance. Thus, for a nonlinear model *M* with model parameters, θ, which need to be estimated from the observed data, including x denoting the system input vector (i.e., the biological input parameters shown in Table 1) and y denoting the output vector (i.e., $G_{H},G_{L},I,\beta$). Let us denote the data D associated to the model, *M* by $D=\{x_{i},y_{i})|i=1,\ldots ,n\}$, where *n* is the number of data points. We also assume the mapping between the input $x_{i}$ to $y_{i}$ can be represented as

y=f(x,θ)+ϵ,

where it is assumed that the observed values y differ from the function values f(x,θ) by additive noise, ϵ, and we will further assume that this noise follows an independent, identically distributed Gaussian distribution with zero mean and variance, $\sigma^{2}$

$$\epsilon ∼N(0,\sigma^{2})$$

The parameters of model *M*, are estimated using the Bayes estimates, which are computed by implementing the MCMC algorithm. The posterior distribution of the parameters, $\left(\theta ,\sigma^{2}\right)$ is given by,

$$p\left(\theta |y,\sigma^{2}\right)\propto \exp(-0.5\times \frac{SS(\theta )}{\sigma^{2}})p_{pri}\left(\theta \right),$$

where *SS* is the sum of squares function ($SS\left(\theta \right)=\sum_{}^{}{\left(y_{i}-f{\left(x,\theta \right)}_{i}\right)}^{2})$ and $p_{pri}\left(\theta \right)$ is the prior distribution of the parameters. A common approach to determine prior distribution for the parameters of the univariate normal model is to assume either a non-informative prior or a conjugate prior distribution for the unknown parameters. We assume a non-informative prior distribution, for θ, as $p_{pri}\left(\theta \right)$, which will be constant for any values of θ. While, for the error precision, $\sigma^{-2}$, as a nuisance parameter, we assume a conjugate prior distribution as a Gamma distribution (adopted from Gelman et al. [50]; see Section 3.4) as follows:

$$p_{pri}\left(\sigma^{-2}\right)∼\Gamma (\frac{n_{0}}{2},\frac{n_{0}}{2}S_{0}^{2})$$

where $n_{0}$ and $S_{0}$ are known, and they can be easily defined in the FME package to compute the posterior distribution for $\sigma^{-2}$. The posterior distribution is calculated using the function *modMCMC*. At each MCMC step, the error variance $\sigma^{-2}$ will be sampled from the following Gamma distribution as the posterior:

$$p\left(\sigma^{-2}|\left(y,\theta \right)\right)∼\Gamma (\frac{n_{0}+n}{2},\frac{n_{0}S_{0}^{2}+SS\left(\theta \right)}{2}).$$

The MCMC chain was generated with at least 100,000 runs for the final model fitting. Chain convergence was examined visually, using the Coda R package. Extended runs were carried out in cases where convergence was not evident. Uncertainty of each estimated parameter was evaluated by analysing the MCMC chains and calculating the 2.5% and 97.5% quantiles to obtain the 95% credible interval (Crls). We used Akaike Information criterion (AIC) [33] and Bayesian information criterion (BIC) [34] to compare and then identify the best model that described blood glucose concentration levels in a type 1 diabetic. We used two types of information criteria to confirm the consistence of results. The AIC evaluated the relative fit of the models given by calculating a prediction error, using the following formula.

$$\mathrm{AIC}=n\log\left(\frac{\hat{L}}{n}\right)+2k.$$

where *n* represents the number of data points used, *k* is the number of parameters fitted (which, for our fitting, is 2 and 4, respectively, for Model 1 and Model 2), and $\hat{L}$ is the maximized value of the likelihood function of the model. BIC also evaluates the prediction error of the models using a different penalty term for increased parameters involved and is given by the following formula.

$$\mathrm{BIC}=k\ln\left(n\right)-2\ln\left(\hat{L}\right).$$

where *n* represents the number of data points used, *k* is the number of parameters estimated by the model (fitted).

Each diabetes model system of differential equations is solved and fitted to experimental data using a code in the R programming environment (with FME and Coda packages) following the algorithm below:

1. Select a dataset and candidate model.
2. Use functions *modFit* and *modCost* to find the best-fit parameters using least squares fit. The function *modFit* conducts constrained fitting of the model to data when fitting a model to data with lower and/or upper bounds. The function *modCost* calculates the discrepancy of a model solution with observed data. This function estimates the residuals, and the variable and model costs (sum of squared residuals), given a solution of the model and data.
3. Use Gaussian likelihood to draw model parameter posteriors for a set of varied parameters (i.e., for each candidate model) assuming uniform non-informative priors.
4. Use the function *modMCMC* to perform MCMC simulations assuming Gaussian likelihood and visually examine chain convergence.
5. Store the model estimates in a table.
6. Compute uncertainty range for each parameter estimate and calculate AIC and BIC values.
7. Plot relevant model output.
8. Repeat steps 1–7 for each dataset and candidate model.

## 3. Results

### Parameter Estimating and Uncertainty

**Figure 2:**
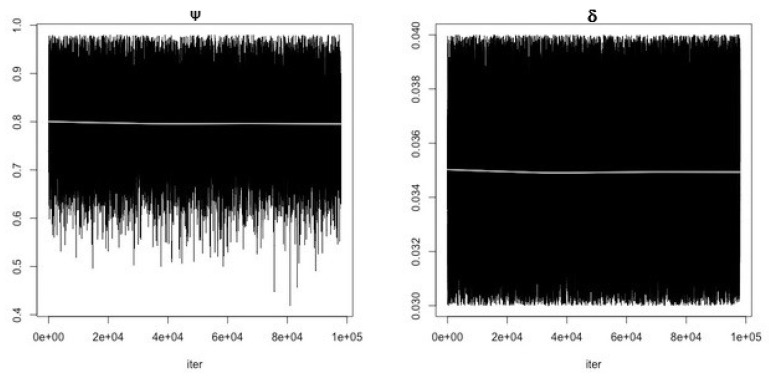
**First panel** shows MCMC chain convergence for parameter ψ and **second panel** shows MCMC chain convergence for parameter δ when fitting Model 1 to Mice Group 1 data. The black lines represent the MCMC chain and the grey line represents traces of the chain.

**Figure 3:**
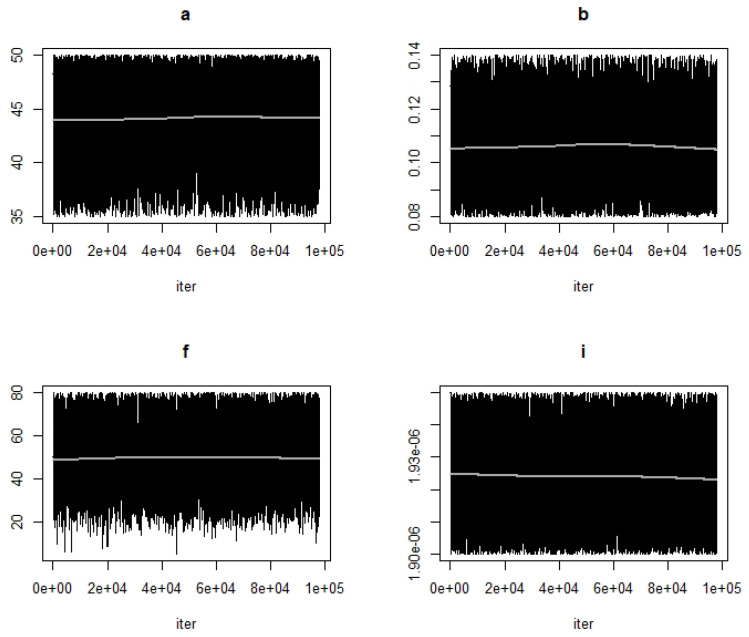
**First top panel** shows MCMC chain convergence for parameter *a* and **second top panel** shows MCMC chain convergence for parameter *b*. The **first bottom panel** shows MCMC chain convergence for parameter *f* and the **second bottom panel** shows MCMC chain convergence for parameter *i*. These MCMC convergences are for Model 2 when fitted to Mice Group 1 data. The black lines represent the MCMC chain and the grey line represents traces of the chain.

**Figure 4:**
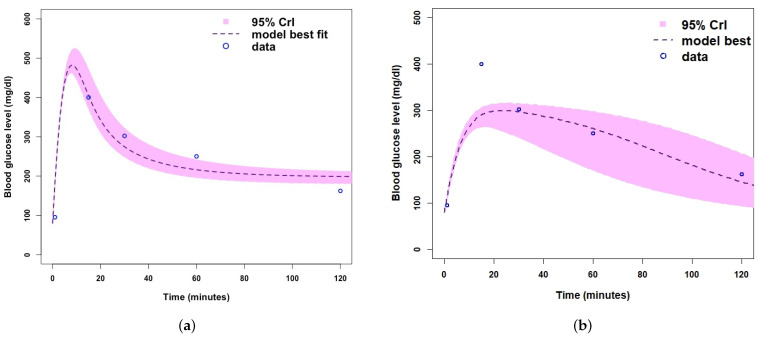
(**a**) Fitting Model 1 (with no β-cells) to blood glucose level dataset for Mice Group 1. (**b**) Fitting Model 2 (with β-cells) blood glucose level dataset for Mice Group 1. Where the shaded light purple region is the 95% Crls, the dashed dark purple line is the median model projection, and the blue circles are the mean data points for the glucose concentration level in Mice Group 1.

**Figure 5:**
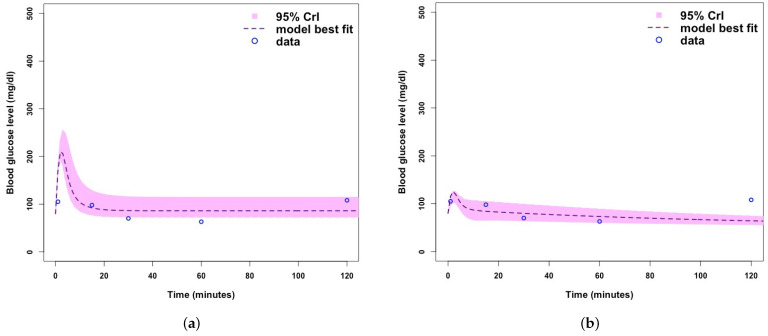
(**a**) Fitting Model 1 (with no β-cells) to blood glucose level dataset for Mice Group 2. (**b**) Fitting Model 2 (with β-cells) to blood glucose level dataset for Mice Group 2. Where the shaded light purple region is the 95% Crls, the dashed dark purple line is the median model projection, and the blue circles are mean data points for the glucose concentration level in Mice Group 2.

**Figure 6:**
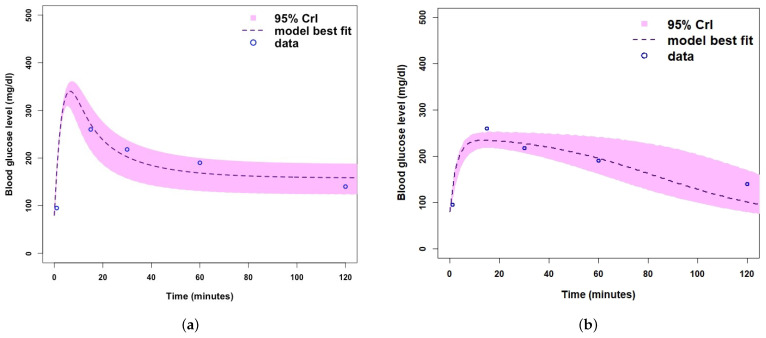
(**a**) Fitting Model 1 (with no β-cells) to Mice Group 3 dataset. (**b**) Fitting Model 2 (with β-cells) to Mice Group 3 dataset. Where the shaded light purple region is the 95% Crls, the dashed dark purple line is the median model projection, and the blue circles are mean data points for the glucose concentration levels in Mice Group 3.

**Figure 7:**
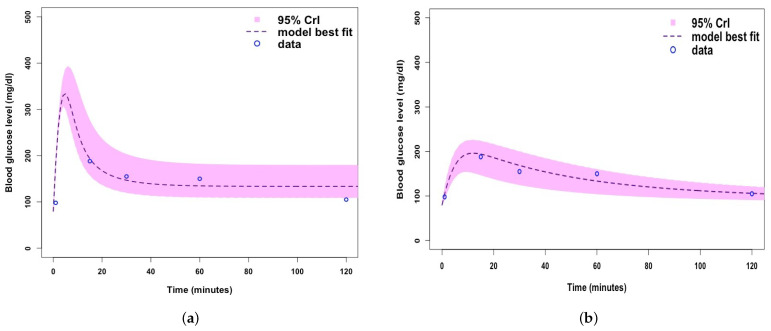
(**a**) Fitting Model 1 (with no β-cells) to blood glucose level dataset for Mice Group 4. (**b**) Fitting Model 2 (with β-cells) to blood glucose level dataset for Mice Group 4. Where the shaded light purple region is the 95% Crls, the dashed dark purple line is the median model projection, and the blue circles are mean data points for the glucose concentration levels in Mice Group 4.

**Figure A1:**
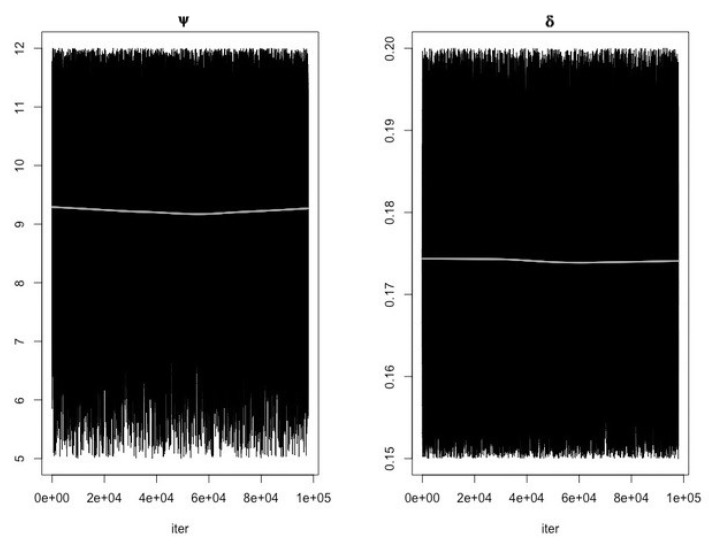
**First panel** shows MCMC chain convergence for parameter ψ and **second panel** shows MCMC chain convergence for parameter δ when fitting Model 1 to Mice Group 2 data. The black lines represent the MCMC chain and the grey line represents traces of the chain.

**Figure A2:**
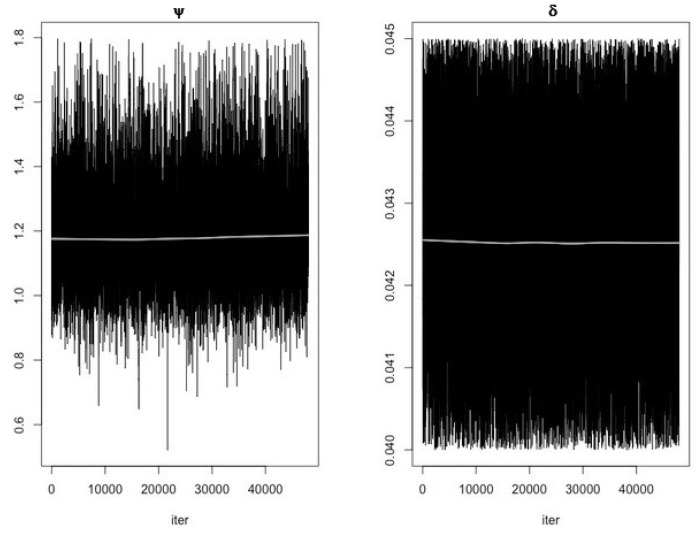
**First panel** shows MCMC chain convergence for parameter ψ and **second panel** shows MCMC chain convergence for parameter δ when fitting Model 1 to Mice Group 3 data. The black lines represent the MCMC chain and the grey line represents traces of the chain.

**Figure A3:**
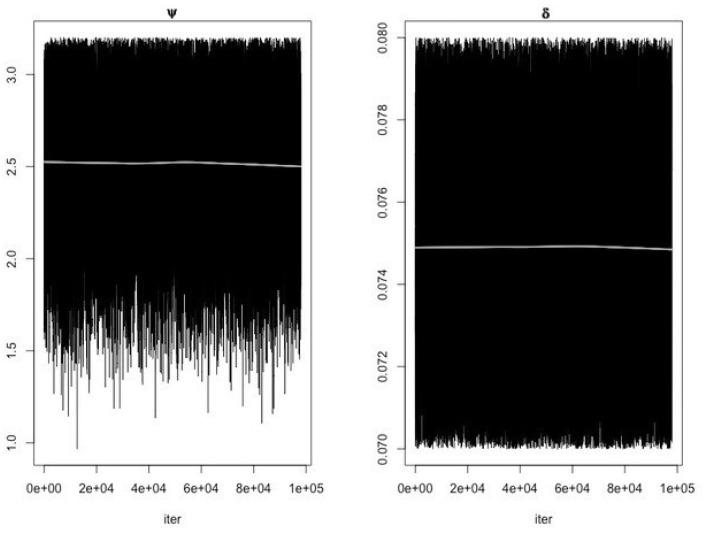
**First panel** shows MCMC chain convergence for parameter ψ and **second panel** shows MCMC chain convergence for parameter δ when fitting Model 1 to Mice Group 4 data. The black lines represent the MCMC chain and the grey line represents traces of the chain.

**Figure A4:**
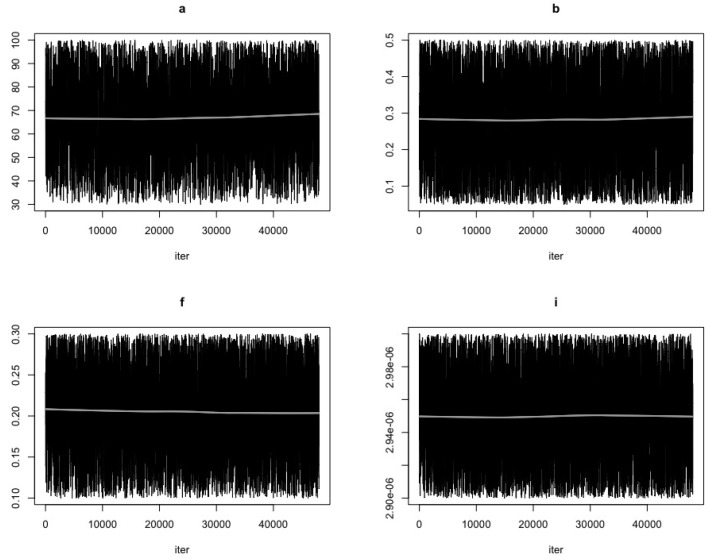
**First top panel** shows MCMC chain convergence for parameter *a* and **second top panel** shows MCMC chain convergence for parameter *b*. The **first bottom panel** shows MCMC chain convergence for parameter *f* and the **second bottom panel** shows MCMC chain convergence for parameter *i*. These MCMC convergences are for Model 2 when fitted to Mice Group 2 data. The black lines represent the MCMC chain and the grey line represents traces of the chain.

**Figure A5:**
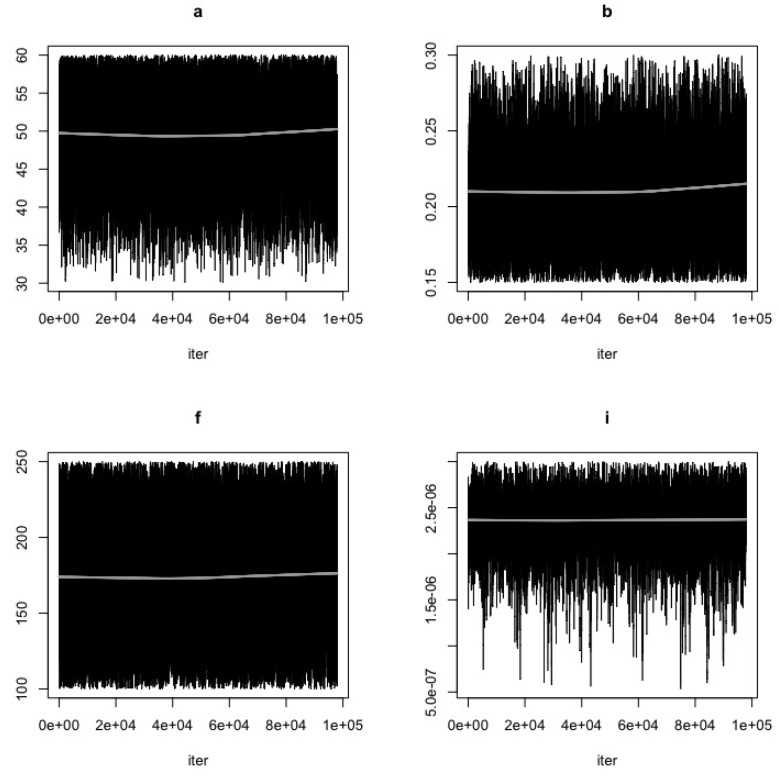
**First top panel** shows MCMC chain convergence for parameter *a* and **second top panel** shows MCMC chain convergence for parameter *b*. The **first bottom panel** shows MCMC chain convergence for parameter *f* and the **second bottom panel** shows MCMC chain convergence for parameter *i*. These MCMC convergences are for Model 2 when fitted to Mice Group 3 data. The black lines represent the MCMC chain and the grey line represents traces of the chain.

**Figure A6:**
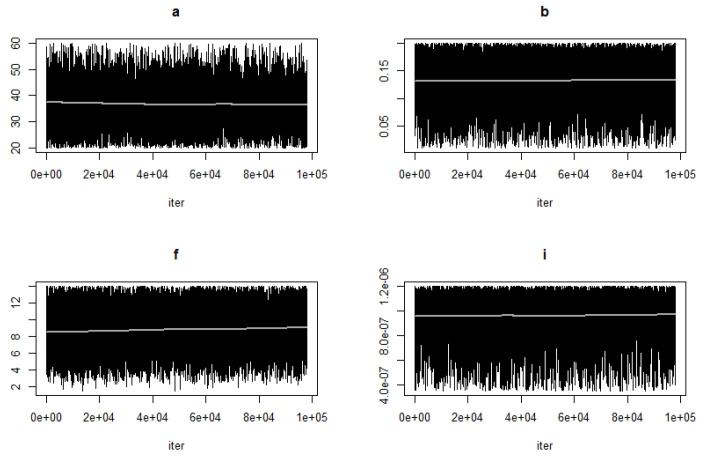
**First top panel** shows MCMC chain convergence for parameter *a* and **second top panel** shows MCMC chain convergence for parameter *b*. The **first bottom panel** shows MCMC chain convergence for parameter *f* and the **second bottom panel** shows MCMC chain convergence for parameter *i*. These MCMC convergences are for Model 2 when fitted to Mice Group 4 data. The black lines represent the MCMC chain and the grey line represents traces of the chain.

MCMC chain convergence when fitting Model 1 to blood glucose concentration levels for Mice Group 1 is shown in Figure 2 and the MCMC chain convergence when fitting to Mice Groups 2–4 data are shown in Appendix A Figure A1, Figure A2 and Figure A3. MCMC chain convergence when fitting Model 2 to blood glucose concentration levels for Mice Group 1 is shown in Figure 3 and the MCMC chain convergence when fitting to Mice Groups 2–4 data are shown in Appendix A Figure A4, Figure A5 and Figure A6. The traces of the MCMC chain (shown by the grey line in the figures) show that the chains have converged (i.e., there is no apparent drift). The results for fitting Model 1 to blood glucose level data for Mice Group 1, 2, 3 and 4 are shown in Figure 4a, Figure 5a, Figure 6a and Figure 7a. The corresponding estimates of the insulin clearance rate (δ) and insulin absorption rate (ψ) are presented in Table 2.

In Table 2, we note that Mice Group 2 has the highest estimates for both δ=0.16 and ψ=8.11. Mice Group 1 has the lowest estimated value of δ among all four groups of mice. We also notice a similar trend for ψ. With Mice Group 1, lower estimates could be a result of having extremely high blood glucose levels, which the systems struggle to control, and this is usually due to lack of insulin or a slow rate of insulin absorption into the blood stream (ψ) and, hence, less insulin needs to be cleared (δ). The results for fitting Model 2 to glucose concentration data for Mice Groups 1, 2, 3, and 4 are presented in Figure 4b, Figure 5b, Figure 6b and Figure 7b. The corresponding estimates for glucose production rate (*a*), glucose clearance rate (*b*), whole body insulin clearance rate (*f*), and glucose tolerance range (*i*) are presented in Table 3.

In Table 3, we note that most of the fitted parameters for Mice Group 3 have the highest estimates, except for *i*, for which Mice Group 2 has the highest estimated value. There is no clear trend for the lowest estimated values, however *b* and *f* both had lowest estimated value for Mice Group 2, at 0.11 and 0.28, respectively. It is interesting to note that the whole body insulin clearance rate (*f*) estimated value varied widely depending on the mouse, with an estimated minimum value of 0.28 and a maximum value of 102.81. The low value estimate for *f* using Mice Group 2 data could be explained by the fact that the mouse is not managing its blood glucose levels and this results in a hypoglycaemic episode, meaning that the mouse is not able to clear insulin quickly. The extreme values of *f* also demonstrate lack of control of the glucose homeostasis system. The results in Table 3 show that the whole body insulin clearance rate (*f*) widely varies depending on the dataset and, therefore, plays a large role in the model fitting.

In Figure 4, we note that when fitting the models to the blood glucose concentration level for Mice Group 1, the predicted model trajectories for Models 1 and 2 peak at approximately 525 mg/dL (a dangerously high level, which, if not reduced immediately, could result in death) and 320 mg/dL (a high value, but not as severe as that predicted for Model 1). Figure 4a, Model 1, shows a sharp and uncontrollable blood glucose level peak, which is expected of a type 1 diabetic. Type 1 diabetics lack control of the glucose homeostasis system, as they have little or no β-cells to contain the rapid rise of glucose concentration levels. Figure 4b shows a controlled blood glucose level peak, which is representative of either a type 1 or type 2 diabetic. The nature of Model 2, with the presence of β-cells makes it more suitable for modelling type 2 diabetes; however, it can also represent type 1 if the system evaluates β cells at zero. It is clear that Model 2 fails to fit high hyperglycaemic data point, which occurs predominately with diabetics, and is not a good fit for representing type 1 diabetic mice. There are several glucose homeostasis models [39,51,52,53,54,55,56,57,58,59]; however, the ones which particularly target type 1 usually use intermolecular dynamics and deal with processes within the molecules. Models that are used to represent glucose homeostasis models for non-diabetic and diabetic (type 2 and type 1) usually use blood concentrations of the glucose and insulin levels, instead of intermolecular dynamics [9]. The advantage of using models with blood glucose and insulin is that they can easily be quantified unlike molecules.

In Figure 5, we fitted the models to blood glucose concentration level data for Mice Group 2. Mice Group 2 is a hypoglycaemic mice group that manages to return its glucose concentration back to normal levels. Model 1 fitting predicts a higher blood glucose peak at approximately 260 mg/dL and Model 2 peaks at approximately 120 mg/dL. This is a significant difference in blood glucose concentration and shows the difference in management of the disease, Model 1 is more representative of an uncontrollable glucose homeostasis system for a Type 1 diabetic. We note that for Model 2 (Figure 5b), the uncertainty range is comparatively very small and fails to fit the final data point, but the model does fit the other data points very well within 95% Crls.

Similarly, in Figure 6, we note that when fitting both mathematical models to the blood glucose concentration level dataset for Mice Group 3, the predicted model trajectories for Models 1 and 2 peak at 370 mg/dL and 265 mg/dL. Both of these are within the feasible range of blood glucose concentration levels [4]. However, Figure 6a shows an extremely high value, as would be expected of type 1 diabetic mice with no control of the glucose homeostasis system. We note that this extreme (and rapid) uncontrollable blood glucose concentration level peak for Model 1 is a good representation of a Type 1 diabetic [60,61]. Comparing Figure 6a,b, we note that the 95% Crls are much smaller for Model 1 than that of Model 2, which shows that there is less variation and uncertainty in fitting Model 1.

The characteristic of Model 1 in predicting rapid blood glucose level peaks is also illustrated in Figure 7. The predicted blood glucose level peak in Figure 7a is similar to that in Figure 5a. The fitting of Model 1 to the Mice Group 4 blood glucose dataset is generally good, regardless of the rapid peak; however, it fails to cover all data points. Model 2 fitting to Mice Group 4 does fit very well and has no rapid uncontrollable peak. Model 2 fits the 95% Crls well as the uncertainty range covers all data points. A comparison of Figure 7a,b shows that Model 2 fits better to blood glucose level data for Mice Group 4.

The computed AIC and BIC values for Model 1 and Model 2 for each mice group are presented in Table 4.

The results in Table 4 show that Model 1 has lower AIC and BIC values when fitting the blood glucose concentration level dataset for all four groups of mice. Fitting Model 1 to glucose concentration data for Mice Group 2 gave the lowest AIC =11.10 and BIC =10.32. The difference for Mice Group 4 ΔAIC and ΔBIC<2 ([62,63]) suggests that Models 1 and 2 are equally capable of explaining the blood glucose concentration data. However the differences in ΔAIC and ΔBIC>2 for Mice Groups 1–3 suggest substantial differences between the models (see Table 4). Both models demonstrated equal capability in predicting blood glucose concentration levels for these data. The lower AIC and BIC values for Model 1 suggest that it is more suitable in predicting blood glucose concentration levels among type 1 diabetics.

**Figure 8:**
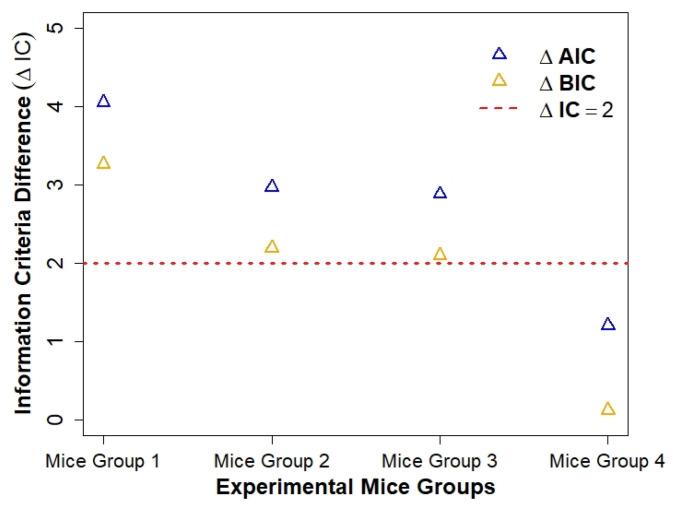
A plot of information criteria difference (ΔIC) for each mice group and corresponding experimental peak blood glucose concentration level. The blue triangles denotes ΔAIC and the orange triangle denotes ΔBIC. The red line denotes ΔIC=ΔAIC or ΔBIC=2.

We notice a general trend in the differences in AIC and BIC results. The magnitude of ΔAIC and ΔBIC values in Table 4 (illustrated in Figure 8) is dependent on the severity of diabetes of each mice group, with increased control of diabetes/decreased severity of diabetes giving lower ΔAIC and ΔBIC<3, and mice with increased severity of diabetes giving higher ΔAIC and ΔBIC>3. This pattern suggests that Model 1 (no β cells) becomes increasingly suitable in explaining blood glucose level data as the mice diabetes condition becomes more severe (i.e., hypoglycaemic and hyperglycaemic episodes are occurring). The severity of diabetes is also increased when the mice progress from type 2 to type 1 diabetes.

## 4. Discussion

Diabetes mismanagement resulting in high blood glucose levels can cause several secondary diseases [64], which could lead to death. Understanding type 1 diabetes and blood glucose concentration levels in individuals at certain significant time points is important in treatment and management of the disease. Mathematical models are important tools to understand pathways and threshold blood glucose concentration levels required to keep the glucose homeostasis system stable. Many of these models are used as algorithms in continuous glucose monitoring devices, insulin pumps, and an artificial pancreas [20,21,22]. The correct insulin bolus injection requires accurate mathematical models in predicting blood glucose concentration levels. Model comparison and selection constitute an important step in determining an accurate model structure for the glucose–insulin regulatory system.

Our results showed that both models (i.e., (with β-cells) and (without β cells)) are comparable in fitting blood glucose level dataset for Mice Group 3 and Mice Group 4. However, the estimated values of ΔAIC=(4.05,2.97) and ΔBIC=(3.26,2.19), for Mice Group 1 and Mice Group 2, blood glucose level datasets showed substantial differences, suggesting that the model without β cells is more suitable in explaining the type 1 glucose homeostasis biological processes. Model fitting (see Figure 4, Figure 5, Figure 6 and Figure 7) showed that the model with no β cells provides a better representation of a type 1 diabetic glucose homeostasis system. The rapid rise in glucose concentration levels simulated in the predictions for the model with no β cells captures a phase in which the system is uncontrollable and requires intervention to be managed, and this is achieved by injecting the insulin bolus [4]. Our findings also showed that, as the mice diabetes condition became more severe, the model with no β cells becomes favourable, as shown by large ΔAIC and ΔBIC values (see Table 4).

Data driven approaches to modelling implemented in this study are important in refining current diabetes models in order to establish evidence based mathematical models with better prediction capabilities and potential to evaluate diabetes management and treatment strategies. The absence of efficient and accurate devices for diabetes treatment and control clearly suggest that current diabetes mathematical models are insufficient. Our study makes an important step in developing a platform for further development of accurate diabetes models using simple deterministic models that are easy to understand.

The study has some limitations. Fitting was only conducted using data for mean blood glucose concentration levels from four groups of mice; however, data for a larger sample of experimental mice would be necessary to fully understand diabetes pathways and performance of the candidate models. It is also important to fit these models to insulin concentration levels data concurrently for more robust results, if such data are available. The prior distributions (non-informative prior for θ and conjugate prior, as inverse Gamma for error variance), considered in the Bayesian framework and introduced in Section 2.3, are appropriate and widely used in the Bayesian studies. However, further investigation is required to examine the robustness of the associated posterior results with respect to prior distributions misspecification. One way to tackle this issue is to formally elicit prior distributions of the parameters, using the methods introduced in [65,66]. However, whilst eliciting prior distributions for the unknown parameters is not very straightforward in practice [67], this is widely recommended as the most appropriate method to conduct a fully Bayesian analysis. An alternative method is to explore sensitivity of the posterior distributions with respect to misspecification of prior distributions. However, local and global sensitivity analyses were common methods from the 1980s to 1990s (see [68,69]), but these methods have recently been further developed and applied in a wide range of applications [70,71,72].

Another limitation is that the data were collected at different time points and it would be interesting to explore the performance of the models for data collected at reduced time intervals or following the significant diabetes time steps (30, 60, 90, 180, and 210 min). These time steps are of relevance, as a diabetic individual would be required to measure their blood glucose levels at 30 and 60 min after food. At 210 min (approximately 3.5 h), the blood glucose levels are expected to be back to normal baseline values as the system achieves homeostasis. The current data only represent an episode of diabetic mice; it is necessary to have continuous data for more episodes in order to develop robust prediction models for type 1 diabetes episodes. The data in [32] explored sex-specific effects of diabetes, it would be interesting to investigate sex-specific effects and disease severity on the performance of the studied models and understand the consistency of our findings. The current model structures could then be modified to include more realistic time dependent parameters (e.g., seasonality terms) or consider using a structure with impulsive differential equations. In addition, it would be interesting to evaluate the predictive capabilities of linear versions of these models, similar to those reviewed in [73], and investigate the structural and practical identifiability [73,74]. Despite these shortfalls, our modelling represents an interesting step in developing data-driven dynamic models for diabetes, to improve the prediction of blood glucose concentrations using deterministic compartmental models.

## 5. Conclusions

This study used data-driven mathematical models to understand diabetes pathways. Mathematical models are important tools in understanding the glucose homeostasis system for diabetic individuals in order to establish disease treatment or management strategies and they are crucial in designing personalized decision support tools for individuals with diabetes [74]. Our findings show that, in general, the candidate model with no β-cells provides a more suitable structure for modelling Type 1 diabetes and predicting glucose concentration for hypoglycaemic episodes.

The findings from this study highlight the need to build suitable mathematical model structures for diabetes. These models are important in developing accurate algorithms for building machine learning predictive models, such as an artificial pancreas [21,22]. Accurate and efficient artificial pancreas machines are essential for diabetes treatment, management, and control. Despite the importance of mathematical models, such as tools to inform decision making for disease (e.g., diabetes), such mathematical models have been neglected for diabetes, with a few mathematical modelling studies on a diabetes homeostasis system available in recent literature. The growing burden of diabetes [1] and complications associated with the COVID-19 infection [5,6], calls for the need to build robust mathematical models—urgently needed tools to control or treat the disease.

## Figures and Tables

**Table 1:** Model parameters, variables, and their definitions. ${}^{*}$ Note that certain parameters are fitted for each model.

Parameter/Variable Definition	Symbol	Baseline Value [Range]	Unit	Reference
**Biological parameters**				
Glucose production rate by liver	*a*	fitted *	mg/dL min	
Glucose clearance rate independent of insulin	*b*	fitted *	min${}^{-1}$	
Insulin induced glucose uptake rate	*c*	0.85 [0.1–1]	mL/m IU min	Assumed
β-cell maximum insulin secretory rate	*d*	43.2 [40–100]	m IU/mL min mg	Assumed
Sigmoidal inflection point	*e*	20,000 [20,000–50,000]	mg${}^{2}$/dL${}^{2}$	Assumed
Whole body insulin clearance rate	*f*	fitted *	min${}^{-1}$	
β-cell natural death rate	*g*	0.03 [0.03–1]	min${}^{-1}$	Assumed
Determines β-cell glucose tolerance range factor	*h*	$0.5727\times 10^{-3}$$[0.5727\times 10^{-3}$–1]	dL/mg min	Assumed
Determines β-cell glucose tolerance range factor	*i*	fitted *	dL${}^{2}$/mg${}^{2}$ min	
Growth hormone production rate by somatotropic cells	ρ	15.06 [5–30]	mIU/mL min	[43]
Growth hormone clearance rate by the liver	*w*	1958.40 [2000–4000]	min${}^{-1}$	[43]
Insulin absorption rate	ψ	fitted *	mIU/mL min	
Insulin clearance rate	δ	fitted *	min${}^{-1}$	
Insulin Bolus	$I_{0}$	5 [5–30]	mIU/mL	[3]
**Model response variables**		**Initial conditions**		
β-cells	β	800	mg	[8]
Insulin	*I*	20	mIU/mL	[44]
Glucose	$G_{L}$	80	mg/dL	[10]
Growth Hormone	$G_{H}$	30	mIU/mL	[45]

**Table 2:** Model 1 parameters estimates with medians and 95% Crls for Mice Groups 1–4. * Note that parameter estimates for δ are multiplied by $10^{-1}$.

Parameter	Mice Group 1	Mice Group 2	Mice Group 3	Mice Group 4
δ$\left(\times 10^{-1}\right)$ *	0.38 (0.3–0.4)	1.6 (1.5–2)	0.42 (0.4–0.45)	0.79 (0.7–0.8)
ψ	0.82 (0.63–0.96)	8.11 (5.67–11.84)	1.21 (0.93–1.60)	2.44 (1.65–3.16)

**Table 3:** Model 2 parameters estimates with medians and 95% Crls for Mice Groups 1–4. * Note that parameter estimates for *i* are multiplied by $10^{-6}$.

Parameter	Mice Group 1	Mice Group 2	Mice Group 3	Mice Group 4
*a*	45.28 (36.02–49.72])	37.29 (34.45–97.24)	86.11 (51.79–97.05)	30.75 (21.21–51.56)
*b*	0.13 (0.08–0.14)	0.11 (0.06–0.49)	0.37 (0.20–0.41)	0.12 (0.03–0.2)
*f*	72.19 (22.26–76.49)	0.28 (0.11–0.3)	102.81 (24.87–243.27)	10.05 (3.77–13.76)
$i\;(\times 10^{-6})$ *	1.91 (1.9–1.95)	2.91 (2.9–3)	2.276 (2.275–2.278)	1.18 (0.51–1.19)

**Table 4:** In the table, ${AIC}_{j}$, ${BIC}_{i}$ for j=1,2 denotes the AIC and BIC values for Models 1 and 2. Similarly $\Delta AIC={AIC}_{2}-{AIC}_{1}$ and $\Delta BIC={BIC}_{2}-{BIC}_{1}$ denotes the difference in AIC and BIC for each model for the same group of mice.

Mouse	${\mathrm{AIC}}_{1}$	${\mathrm{BIC}}_{1}$	${\mathrm{AIC}}_{2}$	${\mathrm{BIC}}_{2}$	ΔAIC	ΔBIC
Mouse 1	11.73	10.95	15.78	14.21	4.05	3.26
Mouse 2	11.10	10.32	14.07	12.51	2.97	2.19
Mouse 3	11.33	10.55	14.21	12.65	2.88	2.10
Mouse 4	11.18	10.70	12.38	10.82	1.20	0.12

## Data Availability

All data in this paper were obtained from published literature in [32].
